# A *SIX6* Nonsense Variant in Golden Retrievers with Congenital Eye Malformations

**DOI:** 10.3390/genes10060454

**Published:** 2019-06-14

**Authors:** Petra Hug, Linda Anderegg, Nicole Dürig, Vincent Lepori, Vidhya Jagannathan, Bernhard Spiess, Marianne Richter, Tosso Leeb

**Affiliations:** 1 Institute of Genetics, Vetsuisse Faculty, University of Bern, 3001 Bern, Switzerland; petra.hug@vetsuisse.unibe.ch (P.H.); linda.anderegg@vetsuisse.unibe.ch (L.A.); nicoleduerig@gmail.com (N.D.); vincent.lepori@gmail.com (V.L.); vidhya.jagannathan@vetsuisse.unibe.ch (V.J.); 2 Ophthalmology Section, Vetsuisse Faculty, University of Zurich, 8057 Zurich, Switzerland; bmspiess@me.com; 3 Eyevet.ch, c/o Tierklinik Aarau West, 5036 Oberentfelden, Switzerland; mrichter@eyevet.ch

**Keywords:** *Canis lupus familiaris*, dog, whole genome sequence, animal model, homeobox, ophthalmology, development, optic nerve, retinal dysplasia, nystagmus

## Abstract

Causative genetic variants for more than 30 heritable eye disorders in dogs have been reported. For other clinically described eye disorders, the genetic cause is still unclear. We investigated four Golden Retriever litters segregating for highly variable congenital eye malformations. Several affected puppies had unilateral or bilateral retina dysplasia and/or optic nerve hypoplasia. The four litters shared the same father or grandfather suggesting a heritable condition with an autosomal dominant mode of inheritance. The genome of one affected dog was sequenced and compared to 601 control genomes. A heterozygous private nonsense variant, c.487C>T, was found in the *SIX6* gene. This variant is predicted to truncate about a third of the open reading frame, p.(Gln163*). We genotyped all available family members and 464 unrelated Golden Retrievers. All three available cases were heterozygous. Five additional close relatives including the common sire were also heterozygous, but did not show any obvious eye phenotypes. The variant was absent from the 464 unrelated Golden Retrievers and 17 non-affected siblings of the cases. The SIX6 protein is a homeobox transcription factor with a known role in eye development. In humans and other species, *SIX6* loss of function variants were reported to cause congenital eye malformations. This strongly suggests that the c.487C>T variant detected contributed to the observed eye malformations. We hypothesize that the residual amount of functional SIX6 protein likely to be expressed in heterozygous dogs is sufficient to explain the observed incomplete penetrance and the varying severity of the eye defects in the affected dogs.

## 1. Introduction

Human congenital eye malformations have been extensively described in the literature. Dominant, recessive and X-linked modes of inheritance have been observed. Some genetic variants exclusively cause eye malformations while others cause syndromic phenotypes with additional modifications beyond the eye [1]. In humans, hereditary eye diseases make up half of the blindness cases in children [2]. Among others, genetic causes for optic nerve hypoplasia [3], congenital cataract [4], and anomalies of the optic disc have been described [5].

Eye diseases in dogs may be caused by different pathogeneses, including neoplasia, trauma, infectious diseases, and genetics [6,7,8,9]. Furthermore, eye changes may occur secondary to systemic diseases such as diabetes mellitus [10] or hypothyroidism [11]. Congenital eye malformations might be caused by a genetic disposition, as well as by exposure to toxins [12] or infections of the mother during pregnancy [13]. In dogs, more than 30 causative genetic variants for heritable eye diseases have been described, most of them leading to various forms of progressive retinal atrophy (PRA), the canine homolog of retinitis pigmentosa in humans (Table 1). Other canine eye disorders have been clinically characterized, but their genetic cause is still unclear [14,15]. Golden Retriever breeders have noticed a new form of ocular malformations in their dogs.

The goal of this study was to characterize the phenotype and identify the underlying causative genetic defect.

## 2. Materials and Methods

### 2.1. Ethics Statement

All animal experiments were performed according to local regulations. All dogs in this study were privately owned and examined with the consent of their owners. The "Cantonal Committee for Animal Experiments" approved the collection of blood samples (Canton of Bern; permit 75/16).

### 2.2. Animal Selection

This study included four Golden Retriever litters, all having the same father or grandfather. In every litter, at least one puppy with eye problems was noted by the breeders. Four puppies with severe eye malformations were euthanized shortly after birth and no samples for genetic analysis were available. Blood samples were collected from 23 offspring (3 affected with eye malformations, 20 non-affected) and 7 non-affected parents including the common male ancestor. Additionally, we used 464 blood samples of Golden Retrievers not closely related to this family, which had been donated to the Vetsuisse Biobank. They represented population controls without reports of ocular malformations.

### 2.3. Clinical Examination

Six cases were presented to two different board-certificated ophthalmologists (BS, MR) at the age of one month to one year. The seventh case had a fatal accident prior to the scheduled eye examination. Eye examinations were performed, including ultrasound examination and post mortem section for some cases (no histological examination was performed). In case 1, an additional magnetic resonance imaging (MRI) examination was done.

### 2.4. DNA Extraction

Genomic DNA was isolated from EDTA blood of 30 members of an extended Golden Retriever family with the Maxwell RSC Whole Blood Kit using a Maxwell RSC instrument (Promega, Dübendorf, Switzerland). Additionally we used DNA from EDTA blood of 459 non-affected control Labrador Retrievers that had been stored in our biobank.

### 2.5. Whole Genome Sequencing of an Affected Golden Retriever

An Illumina TruSeq PCR-free DNA library with 400 bp insert size of an affected Golden retriever (case 7, GR1161) was prepared. We collected 192 millions 2 × 150 bp paired-end reads on a NovaSeq 6000 instrument (22.7× coverage). Mapping and alignment were performed as described [54]. The sequence data were deposited under the study accession PRJEB16012 and the sample accession SAMEA4867924 at the European Nucleotide Archive. 

### 2.6. Variant Calling

Variant filtering was performed as described [54]. To predict the functional effects of the variants, SnpEFF [55] software, together with NCBI annotation release 105 for CanFam 3.1, was used. For variant filtering we used 601 control genomes, which were either publicly available [56] or produced during other projects of our group (Supplementary Table S1).

### 2.7. Gene Analysis

We used the dog CanFam 3.1 reference genome assembly for all analyses. Numbering within the canine *SIX6* gene corresponds to the NCBI RefSeq accessions XM_547840.6 (mRNA) and XP_547840.3 (protein).

### 2.8. Sanger Sequencing

The *SIX6*:c.487C>T variant was genotyped by direct Sanger sequencing of PCR amplicons. A 521 bp PCR product was amplified from genomic DNA using AmpliTaqGold360Mastermix (Thermo Fisher, Basel, Switzerland together with primers 5′-CCG CGA GCT CTA CCA TAT TC-3′ (Primer F) and 5′-AAC GCA GTG GGC TTG TAA CT-3′ (Primer R). After treatment with exonuclease I and alkaline phosphatase, amplicons were sequenced on an ABI 3730 DNA Analyzer (Thermo Fisher). Sanger sequences were analyzed using the Sequencher 5.1 software (GeneCodes, Ann Arbor, MI, USA.

## 3. Results

### 3.1. Eye Examinations

**Figure 1:**
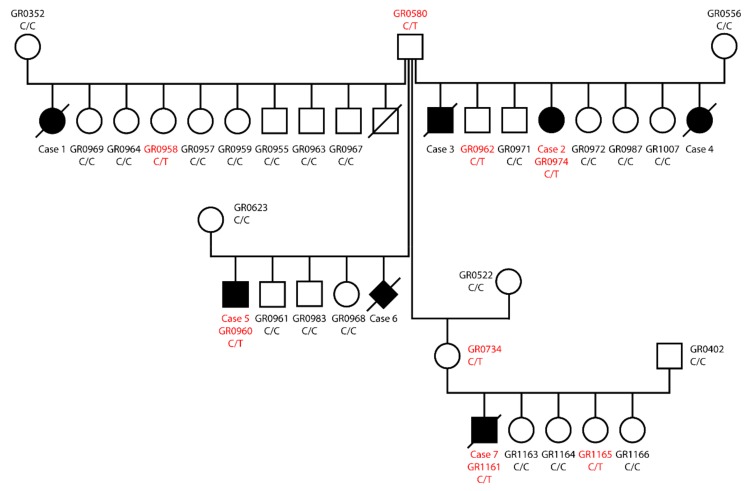
Pedigree of the four litters investigated. Filled symbols represent dogs with eye malformations. Open symbols represent dogs in which no eye abnormalities were observed by their owners. These dogs were not specifically examined by an ophthalmologist (or the results of such an examination were not available for the study). Deceased or euthanized animals are indicated by strikethrough symbols. *SIX6*:c487C>T genotypes are indicated for all dogs, from which a blood sample was available. Heterozygous C/T genotypes are indicated in red. Please note that all three available cases, but also five dogs with no apparent eye phenotypes, carried the mutant T-allele.

Four litters with the same father or grandfather contained at least one puppy with eye malformations. A total of seven puppies with eye malformations were investigated. The manifestations were quite variable and were present unilaterally or bilaterally. The parents and siblings showed no clinical signs of eye malformations. The pedigree of the four litters is shown in Figure 1. The clinical findings are summarized in Supplementary Table S2.

Case 1 was presented due to congenital nystagmus and visual impairment. No reduced reflexes were observed in the left eye, the anterior segment of the eye appeared normal but hypoplasia of the optic nerve was documented. The right eye showed few reflexes, mydriasis, a congenital cataract with posterior lenticonus, retinal dysplasia with retinal detachment and also a hypoplasia of the optic nerve. Following the eye examination, an MRI of the skull and a cerebrospinal fluid puncture were performed. A malformation of the cinguli gyrus at the transition between the third and fourth ventricle was observed. No changes in the liquor were detected. The puppy had to be euthanized due to aggressivity.

Case 2 was presented due to a one-sided opacity of the left eye. The right eye was without special findings. The left eye showed an extensive persistent pupillary membrane (lamina) from the iris to the cornea nasally, the anterior chamber of the eye was clear, the lens was clear but no good view of the fundus was given. Since the menace reaction can only be tested approximately from the 4th month of life and this puppy was examined at 6 weeks of age, no statement about the function of the optic nerve could be made. Later, blindness was observed in this dog in the left eye. The right eye is still without special findings to our knowledge.

Case 3 was presented due to conspicuous behavior. The puppy bumped into objects and could not find his way around well. The eyes showed a clouding on both sides. In both eyes, a large nasally located persistent pupillary membrane (lamina) was seen. In the right eye, thick white convoluted folds with some blood vessels were found at the posterior capsule, which lead the suspicion of a retinal ablation or remnants of embryonic tissue retrolental. In the left eye, there was a whitish veil behind the lens, which made the view of the retina impossible. Ultrasound examination revealed a deformed lens on the right side with dense hyper echogenic irregular deposits on the posterior lens capsule, leading to the suspicion of a persistent hyperplastic tunica vasculosa lentis and possibly an additional dysplastic retina. No *arteria hyaloidea* was recognizable. In the left eye, retrolental transverse echogenic structures were present in the vitreous body, which resonated with globe movement. A retinal detachment was suspected. The puppy was euthanized due to these findings and the poor prognosis. The eye was removed for macroscopic post-mortem evaluation. No papilla or optic nerve strand was found.

Case 4 was presented due to conspicuous behavior. The puppy bumped into objects and could not find his way around well. The eyes showed a clouding on both sides. In both eyes, a large nasally located persistent pupillary membrane (lamina) was seen. On both sides, there were streaks in the vitreous body and the fundus was not assessable. The ultrasound examination did not reveal clear changes in the area of the lens and retina. However, the macroscopic evaluation following euthanasia revealed bilateral complete absence of retina, papilla and optic nerve.

Case 5 demonstrated behavioral abnormalities. The left eye was inconspicuous. A retinal detachment or retinal dysplasia was detected in the right eye.

Case 6 exhibited behavioral abnormalities. Large-area laminar synechia in the nasal chamber angle in the right eye were seen. In the left eye, a missing optic nerve and retinal dysplasia with abnormal vascular pattern was diagnosed. This puppy was euthanized due to bilateral changes.

Case 7 showed spontaneous nystagmus since birth and progressive ataxia with increasing age. The dog suffered a fatal car accident prior to the scheduled eye examination.

### 3.2. Genetic Analysis

All cases traced back to the same male ancestor, but had quite diverse maternal lineages (Figure 1). Seven out of 28 recorded puppies were affected and males and females were affected in equal proportions. As we could not identify any common ancestors in the maternal lines of the cases, we hypothesized an autosomal dominant inheritance with incomplete penetrance as the most likely mode of inheritance.

We sequenced the genome of case 7 at 22.7× coverage and called SNVs and short indels with respect to the canine reference genome assembly CanFam 3.1. We then searched for heterozygous and homozygous variants in the genome sequence of the affected dog that were not present in 601 control dogs of different breeds (Table 2).

We identified 21 private protein-changing variants (Table S2). We prioritized these variants based on known functions of the respective genes/proteins from the literature. Based on this prioritization, we considered a nonsense variant in *SIX6* the most likely candidate causative variant as variants in the *SIX6* gene lead to comparable eye defects in humans and mice [57,58,59,60].

The variant was located in the first exon of the *SIX6* gene and can be designated as Chr8:35,566,504C>T (CanFam 3.1 assembly). This variant, XM_547840.6:c.487C>T, introduced a premature stop codon and was predicted to truncate 84 of the 246 codons of the *SIX6* open reading frame (XP_547840.3:p.(Gln163*)).

**Figure 2:**
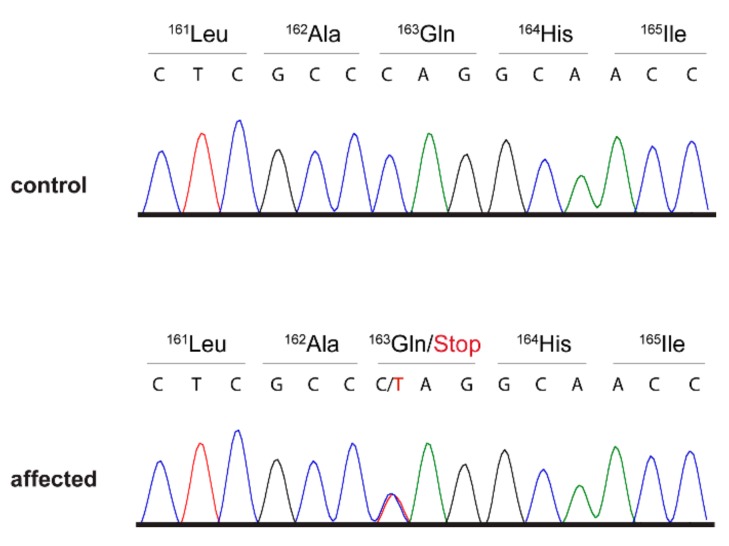
Details of the *SIX6:*c.487C>T variant. Representative electropherograms of two dogs with different genotypes are shown. The variable position is indicated by an arrow.

We confirmed the presence of the *SIX6* variant by Sanger sequencing and genotyped all available family members (Figure 1, Figure 2). All three available cases were heterozygous at the variant. The non-affected common sire and four additional non-affected family members were also heterozygous. The mutant allele was absent from 464 additionally genotyped Golden Retrievers that were not closely related to the investigated family (Table 3).

## 4. Discussion

The c.487C>T variant identified in three dogs with congenital eye malformations affects the *SIX6* gene encoding a well-known homeobox transcription factor [61]. Homeobox transcription factors play an important role in the development of many different organ systems [62]. Homeobox genes are highly conserved from invertebrates to vertebrates [63]. The homeobox genes of animal genomes can be divided into 11 classes, one of them being the SINE class, named after the *Drosophila* gene *sine oculis*, which is essential for the correct development of the visual system in *Drosophila* [64]. The vertebrate SINE class contains six genes (*SIX1-6*). They encode proteins with a SIX domain and a homeodomain. [65]. The *sine oculis* vertebrate homologs *SIX3* and *SIX6* play an important role in the development of the optical system in the most cranial segment of the embryo [66,67].

In humans, a complete loss of *SIX6* function has been reported to cause optic disc anomalies, microphthalmia or anophthalmia [57,58]. *SIX6* haploinsufficiency was suggested to be the cause of bilateral anophthalmia and pituitary anomalies [59,60]. A heterozygous SIX6:p.Thr165Ala substitution was found in a human patient with congenital bilateral asymmetric microphthalmia, cataract and nystagmus. This patient had inherited the mutant allele from her clinically normal father, suggesting incomplete penetrance for this variant [60]. The human phenotype has also been termed “optic disc anomalies with retinal and/or macular dystrophy (ODRMD)”, OMIM number #212550. *Six6* knockout mice are characterized by retinal and pituitary hypoplasia [67].

The extensive knowledge about the function of *SIX6* in mammalian eye development strongly supports the hypothesis that heterozygosity at c.476C>T is indeed the genetic cause for the observed eye malformations in the studied Golden Retriever family. The imperfect genotype-phenotype association suggests incomplete penetrance of this variant. This seems a plausible scenario, as heterozygous dogs should still express some functional wildtype SIX6 protein.

Unfortunately, several of the affected puppies were euthanized without taking blood and/or tissue samples for a more detailed investigation. Therefore, no tissue samples for experimental analysis of the consequences on the transcript or protein level were possible. The premature stop codon generated by the c.487C>T variant is 85 nucleotides upstream of the end of exon 1. As the *SIX6* gene has only two exons, it cannot be ruled out that at least a fraction of the mutant transcript escapes nonsense mediated decay (NMD) and is actually translated into protein. The predicted mutant protein would consist of the SIX6 N‑terminus and a partial homeodomain. While it is unlikely that such a severely truncated mutant protein might act as a completely functional transcription factor, it might still be able to dimerize with wildtype SIX6 and thus have a dominant-negative effect. Alternatively, *SIX6* haploinsufficiency might also be responsible for the phenotype, similar to what has been observed in some human patients with heterozygous *SIX6* variants [59,60].

The observed incomplete penetrance might be explained with minor variations in the actual amount of functional SIX6 protein in heterozygous dogs. Such variation in residual SIX6 expression might be controlled by additional genetic and/or environmental factors.

To the best of our knowledge, this is the first time that a *SIX6* variant was reported in dogs with eye malformations. These dogs represent a potential model for the homologous human developmental defect. Genetic testing can now be implemented. Given the severe phenotype in some of the cases, we recommend that carriers of the mutant allele should be excluded from breeding. As the variant is still very rare in the general Golden Retriever population, this will not cause a major loss of genetic diversity.

## 5. Conclusions

We identified the *SIX6*:c.487C>T nonsense variant as candidate causative variant for congenital eye malformations in Golden Retrievers. The mutant allele most likely acts in a dominant mode of inheritance with incomplete penetrance.

## Figures and Tables

**Table 1:** Overview on canine inherited eye diseases.

Gene	Phenotype	Inheritance	Breed	OMIA	Ref.
*ABCA4*	Stargardt disease 1	AR	Labrador Retriever	002179-9615	[16]
*ADAM9*	cone-rod dystrophy 3	AR	Glen of Imaal Terrier	001520-9615	[17]
*ADAMTS10*	POAG	AR	Beagle, Nor. Elkhound	001870-9615	[18,19]
*ADAMTS17*	POAG and/or PLL	AR	many	001976-9615	[20,21]
*BEST1*	multifocal retinopathy 1	AR	many	001311-9615	[22]
*BEST1*	multifocal retinopathy 2	AR	Coton de Tulear	001553-9615	[22]
*BEST1*	multifocal retinopathy 3	AR	Lapponian Herder	001554-9615	[23]
*CCDC66*	generalized PRA	AR	Schapendoes	001521-9615	[24]
*CNGA1*	PRA	AR	Shetland Sheepdog	001977-9615	[25]
*CNGB1*	PRA	AR	Papillon	000830-9615	[26]
*CNGB3*	achromatopsia	AR	many	001365-9615	[27]
*COL9A2*	oculoskeletal dysplasia 2	AR	Samoyed	001523-9615	[28]
*COL9A3*	oculoskeletal dysplasia 1	AR	Labrador Retriever	001522-9615	[28]
*FAM161A*	PRA, type 3	AR	several	001918-9615	[29]
*HSF4*	cataract, early onset	AR	many	001758-9615	[30]
*IQCB1*	cone-rod dystrophy 2	AR	Am. Pit Bull Terrier	001675-9615	[31]
*MERTK*	PRA	AR	Swedish Vallhund	001932-9615	[32]
*NECAP1*	PRA	AR	Giant Schnauzer	n.a.	[33]
*NHEJ1*	Collie eye anomaly	AR	many	000218-9615	[34]
*NPHP4*	cone-rod dystrophy	AR	Dachshund	001455-9615	[35]
*OLFML3*	goniodysgenesis	AR	Border Collie	001223-9615	[36]
*PCARE*	rod-cone dysplasia 4	AR	many	001575-9615	[37]
*PDE6A*	rod-cone dysplasia 3	AR	Cardigan Welsh Corgi.	001314-9615	[38]
*PDE6B*	cone-rod dystrophy 1	AR	Am. Staff. Terrier	001674-9615	[31]
*PDE6B*	rod-cone dysplasia 1	AR	Irish Setter	000882-9615	[39]
*PDE6B*	rod-cone dysplasia 1a	AR	Sloughi	001669-9615	[40]
*PPT1*	photoreceptor dysplasia	AR	Miniature Schnauzer	001311-9615	[41]
*PRCD*	prog. rod-cone degeneration	AR	many	001298-9615	[42]
*RD3*	rod-cone dysplasia 2	AR	Collie	001260-9615	[43]
*RHO*	autosomal dominant PRA	AD	Bull & English Mastiff	001346-9615	[44]
*RPE65*	Leber congenital amaurosis	AR	Briard	001222-9615	[45]
*RPGR*	RRA, X-linked, type 1	X-linked	many	000831-9615	[46,47]
*RPGR*	PRA, X-linked, type 2	X-linked	mixed breed dog	001518-9615	[46]
*RPGRIP1 *(&* MAP9*)	cone-rod dystrophy 4	complex	Dachsund	001432-9615	[48,49]
*SAG*	PRA	AR	Basenji	001876-9615	[50]
*SLC4A3*	Golden Retriever PRA 1	AR	Golden Retriever	001572-9615	[51]
*STK38L*	early retinal degeneration	AR	Norwegian Elkhound	001297-9615	[52]
*TTC8*	Golden Retriever PRA 2	AR	Golden Retriever	001984-9615	[53]

**Table 2:** Results of variant filtering.

Filtering Step	Heterozygous Variants	Homozygous Variants
variants in the whole genome	3,024,455	2,913,164
private variants	8983	1214
protein-changing private variants	19	2

**Table 3:** Genotype phenotype association of the *SIX6:*c.487C>T variant.

Dogs	C/C	T/C
Cases (*n* = 3)	0	3
Non-affected family members (*n* = 22)	17	5
“unrelated>” Golden Retrievers (*n* = 464)	464	0

## Supplementary Materials

The following are available online at https://www.mdpi.com/2073-4425/10/6/454/s1, Table S1: Accession numbers of 594 dog and 8 wolf genome sequences, Table S2: Summary of the clinical findings in the 7 examined Golden Retrievers with eye malformations, Table S3: Private protein changing variants in the sequenced Golden Retriever with eye malformation.
